# Corpus callosum long-term biometry in very preterm children related to cognitive and motor outcomes

**DOI:** 10.1038/s41390-023-02994-4

**Published:** 2024-01-15

**Authors:** Manuel Lubián-Gutiérrez, Isabel Benavente-Fernández, Yolanda Marín-Almagro, Natalia Jiménez-Luque, Amaya Zuazo-Ojeda, Yolanda Sánchez-Sandoval, Simón P. Lubián-López

**Affiliations:** 1 Division of Neurology, Department of Paediatrics, Puerta del Mar University Hospital, Cádiz, Spain; 2 Area of Paediatrics, Department of Child and Mother Health and Radiology, Medical School, University of Cádiz, C/Doctor Marañón, 3, Cádiz, Spain; 3 Biomedical Research and Innovation Institute of Cádiz (INiBICA) Research Unit, Puerta del Mar University Hospital, Cádiz, Spain; 4 Division of Neonatology, Department of Paediatrics, Puerta del Mar University Hospital, Cádiz, Spain; 5 Radiology Department, Puerta del Mar University Hospital, Cádiz, Spain; 6 Area of Developmental and Educational Psychology, Department of Psychology, University of Cádiz, Cádiz, Spain

## Abstract

**Background:**

The corpus callosum (CC) is suggested as an indirect biomarker of white matter volume, which is often affected in preterm birth. However, diagnosing mild white matter injury is challenging.

**Methods:**

We studied 124 children born preterm (mean age: 8.4 ± 1.1 years), using MRI to assess CC measurements and cognitive/motor outcomes based on the Wechsler Intelligence Scale for Children-V (WPPSI-V) and Movement Assessment Battery for Children-2 (MABC-2).

**Results:**

Children with normal outcomes exhibited greater height (10.2 ± 2.1 mm vs. 9.4 ± 2.3 mm; *p* = 0.01) and fractional anisotropy at splenium (895[680–1000] vs 860.5[342–1000]) and total CC length (69.1 ± 4.8 mm vs. 67.3 ± 5.1 mm; *p* = 0.02) compared to those with adverse outcomes. All measured CC areas were smaller in the adverse outcome group. Models incorporating posterior CC measurements demonstrated the highest specificity (83.3% Sp, AUC: 0.65) for predicting neurological outcomes. CC length and splenium height were the only linear measurements associated with manual dexterity and total MABC-2 score while both the latter and genu were related with Full-Scale Intelligence Quotient.

**Conclusions:**

CC biometry in children born very preterm at school-age is associated with outcomes and exhibits a specific subregion alteration pattern. The posterior CC may serve as an important neurodevelopmental biomarker in very preterm infants.

**Impact:**

- The corpus callosum has the potential to serve as a reliable and easily measurable biomarker of white matter integrity in very preterm children.
- Estimating diffuse white matter injury in preterm infants using conventional MRI sequences is not always conclusive.
- The biometry of the posterior part of the corpus callosum is associated with cognitive and certain motor outcomes at school age in children born very preterm.
- Length and splenium measurements seem to serve as reliable biomarkers for assessing neurological outcomes in this population.

## Introduction

Corpus callosum (CC) is the largest telencephalic commissural tract and biggest white matter structure in the human central nervous system. It is present in placental mammals and constitutes the highest level of neocortical interhemispheric connection.^1^ Bundles of axonal fibers that cross midline forming the CC are paramount to integrate lateralized sensory-motor tasks.^2^ Taking into account the extensive connections of very diverse cortical areas (sensory, motor, integration areas…), it has been shown that the biometry and microarchitecture of the CC is related to cognitive and motor functions. As the most important human commissural tract, CC measurements on midsagittal plane could serve as surrogate markers to estimate total cerebral white matter volume in children with white matter diseases.^3–6^

Preterm birth is a major worldwide health problem.^7^ In the past three decades survival of preterm infants has increased and, even though moderate to severe brain injury has been reduced, the risk of neurodevelopmental impairment remains high for those preterm infant born before 32 weeks of gestation.^8–10^ White matter injury (WMI) is the most prevalent brain injury in very preterm infants.^11,12^ It is considered as a spectrum of neuropathological injuries including: focal cystic necrosis, punctate and focal microscopic necrosis and diffuse non-necrotic lesions.^13^ Up to 50% of very preterm infants (VPT) show some degree of WMI on magnetic resonance imaging (MRI)^14,15^ with diffuse WMI being the most prevalent form.^16^ MRI is the most sensitive tool for WMI detection in infants,^17–19^ however conventional MRI sequences at term equivalent age (TEA) are not able to detect mild forms of diffuse WMI. Moreover, some postmortem studies have shown that up to 82% of periventricular leukomalacia have only microscopic necrotic foci.^20^

CC measurements have been widely used in WMI scoring classifications. Whether by MRI or ultrasound, most of them include CC measurements in their scales (mostly thinning of CC).^17,21,22^ The pattern of CC development throughout childhood has been proven to be different in those born preterm from their peers at term, related to gestational age (GA) at birth.^23–26^ However, there is still no consensus on what the best approach to CC biometry as a biomarker for long-term neurodevelopmental outcomes is, and many classifications and subdivisions of the CC have been proposed according to its anatomy and function.^27^ Many of these subdivisions are not easily replicable, are time-consuming and require segmentation post-processing algorithms not available in all hospitals. Complementing conventional sequences, diffusor tensor imaging (DTI) provides a new insight into the microstructural characteristic of brain structures. DTI is based on measuring spatial diffusion of water molecules along the white matter tracts. Quantitative parameters like fractional anisotropy (FA), which is considered a biomarker related to myelination, allow the study of the differential maturational process that occur in children who were preterm compared to those who were born at TEA.^28–31^ Previous studies using DTI have linked lower CC thickness in preterm infants with abnormal bundles.^31^

Our aim is to study the CC biometry and FA in school-age children (aged 6–11) who were VPT, and their association with neurodevelopmental motor and cognitive outcomes.

## Methods

### Participants

This is a prospective observational cohort study including school-age children who were born at a GA equal to or less than 32 weeks and/or with a birth weight equal to or less than 1500 g, admitted to the Neonatal Intensive Care Unit (NICU) in Puerta del Mar University Hospital, Cadiz, Spain. We included a retrospective analysis of the MRI performed at TEA. This study was reviewed and approved by the local Research and Ethics Committee and with parental or legal guardian signed informed consent.

Exclusion criteria were chromosomal or genetic identified anomalies, proven metabolic or malignant disorders, congenital neurological malformation, and congenital infections.

Those children born preterm included, with a mean age of 8.3 years old, were assessed from January 2020 to December 2022. We performed MRI, motor and neuropsychological assessments. Perinatal, neonatal clinical course and neuroimaging data were collected retrospectively.

Socioeconomical status (SES) was measured using maternal level of education at the time of recruitment^32,33^ which was then categorized into three groups according to the number of years of maternal education: primary or secondary school, undergraduate degree, or postgraduate degree (low, medium and high, respectively). Perinatal and postnatal variables were prospectively collected. We considered moderate to severe bronchopulmonary dysplasia if there was need for supplemental oxygen and/or positive pressure at 36 weeks postmenstrual age; significant patent ductus arteriosus if requiring surgical or pharmacological closure; late onset sepsis in the presence of systemic signs of infection and isolation of a bacterial pathogen in blood culture after the first 72 h of life; confirmed necrotizing enterocolitis (Bell Stage II or higher); and severe retinopathy of prematurity (stage 3 or higher).

### Magnetic resonance imaging

We performed an MRI at elementary school age (6–11 years) on the included subjects, and we retrieved, when available, the TEA-MRI that was performed as part of the standard clinical practice at the time.

MRI scans were performed using the Magnetom Symphony 1.5 T scanner (Siemens Health Care, Erlangen, Germany) located in the radiology unit. MRI scans included T1-weighted volumetric images (Multiplanar Reconstruction (MPR)) acquired using the 1.5 Tesla scanner (slice thickness 1.0 mm; echo-time 3.53 ms; flip angle 15°; field of view 192 × 256 mm^2^), axial spin echo T2-weighted images and diffusion tensor imaging (DTI). Every MRI was studied by an experienced radiologist who classified the findings as abnormal if there was evidence of brain injury or normal/mild abnormalities. Neuroimaging measures were performed using Carestream (© 2019 Carestream Health, Version 12.1.5.5151) and CC biometry was assessed using MPR T1-weighted sequences. DTI was acquired with a multirepetition, single-shot echo planar sequence with 120 gradient directions (TR, 3100; TE, 98; flip angle 90; no gap), and diffusion weighting of 1000 s/mm2 (b value) and an image without diffusion weighting, resulting in an in-plane resolution of 1.3 mm. FA ranges from 0 (indicating isotropic diffusion of water molecules) to 1 (anisotropic), and it rises with the development of white matter microstructure.^34^ Mean FA values were obtained from 2 manually selected CC regions of interest (genu and splenium of the CC) on one selected axial plane of the color FA maps (Fig. 2).

MRI at TEA was evaluated using the scale published by ref. ^21^ This classification assesses development and injury of cortical and deep gray matter, white matter, and cerebellum. The overall score obtained classifies MRI findings as: normal (0–3 points) or abnormal: mild (4–7 points), moderate (8–11 points) and severe (≥12 points).

### Corpus callosum measurements

We measured, according to ref. ^35^, five linear parameters in a T1-weighted midline sagittal section: CC length as the distance between the most anterior aspect of the genu and the most posterior aspect of the splenium. Thickness measurements of the CC at different levels (genu, body, isthmus and splenium) were taken tracing a line from the bottom to the top, perpendicular to the curvilinear axis of CC (Fig. 2). All these linear measurements were expressed in millimeters.

We assessed the total midsagittal area of the CC and performed a subdivision of this total area into five sections according to the studies by Witelson,^36^ Duara,^37^ Hofer,^38^ Delacoste,^39^ Shin^40^ and Westerhausen.^41^ In order to do this, CC length was divided in 5 sections and then tracing upward perpendicular lines. Anterior and posterior areas count as one-fifth of total CC area, respectively. The central area is the part in between both anterior and posterior, three-fifths of the total CC area. Areas were expressed in square millimeters.

### Neurodevelopmental outcomes

Cognitive function was evaluated using the Wechsler Intelligence Scale for Children—Fifth Edition (WISC-V).^42^ This scale provides tests and composite scores (indices) reflecting intellectual functioning in five specific domains (working memory, verbal comprehension, visuospatial, processing speed, and fluid reasoning) and Full-Scale Intelligence Quotient (FSIQ), a standardized composite score with a mean of 100 and a standard deviation (SD) of 15. A cut-off value of 85 was considered to further classify participants in groups of normal versus borderline/stablished intellectual disability.

Motor abilities were evaluated using the Movement Assessment Battery for Children, Second Edition (MABC-2)^43^ and divided in 3 domains: manual dexterity, aiming and catching, and balance. The sum of the scores of three domains gives a total MABC-2 punctuation and percentile score. A total MABC-2 percentile score of 15 or over is considered normal. A percentile score of under 15 is considered a risk of movement disability and under 5 is considered diagnostic for developmental coordination disorder.^42^

Children with cerebral palsy with a Gross Motor Functional Classification System level II to III that were not able to complete all the subtests of motor testing would be considered to score under 5 on the MABC-2 total score and have an imputed percentile value of 0.1, according to similar studies.^44^

We considered a global adverse outcome in those children who scored under the 15th centile in MABC-2 total percentile score and/or had a FSIQ score of less than 85.

### Statistical analysis

Quantitative variables were described using the median (Md) or mean value and interquartile range [IQR] or SD, according to their distribution. Bivariate analysis was performed using Pearsonʼs chi-squared test or Fisherʼs exact test for categorical data, and Studentʼs *t*-test or Mann–Whitney *U* test for continuous variables.

Multivariable linear and logistic regression models were used on perinatal variables. We performed multivariable regression and logistic models to study the relationship between CC measurements and neurodevelopmental outcome, including perinatal variables (GA, birth weight and sex), and age at scan. Variables were selected based on the theoretical background and results of bivariate analysis.

Moreover, to allow for non-hierarchical models, we also selected the best logistic model by using the method of all possible equations, which identified the best subset for logistic regression based on all the possible combinations of independent variables (linear measurements of CC and areas, adjusting by birth weight, GA and sex). For each subset area under the curve (AUC), Akaike information criterion (AIC), Schwarz Bayesian Criterion (BIC), sensitivity and specificity were studied as goodness-of-fit measures. Internal validation of the model was performed with cross-validation on the specified models in order to evaluate the model’s ability to fit out-of-sample data. Standardized beta coefficients were estimated to compare the effect size of the independent variables.

Statistical analysis was conducted using Stata 17.0 (Stata Statistical Software: Release 17. College Station, TX: StataCorp LP). A result was considered statistically significant at *p* < 0.05.

## Results

### Clinical characteristics and long-term outcome

**Fig. 1:**
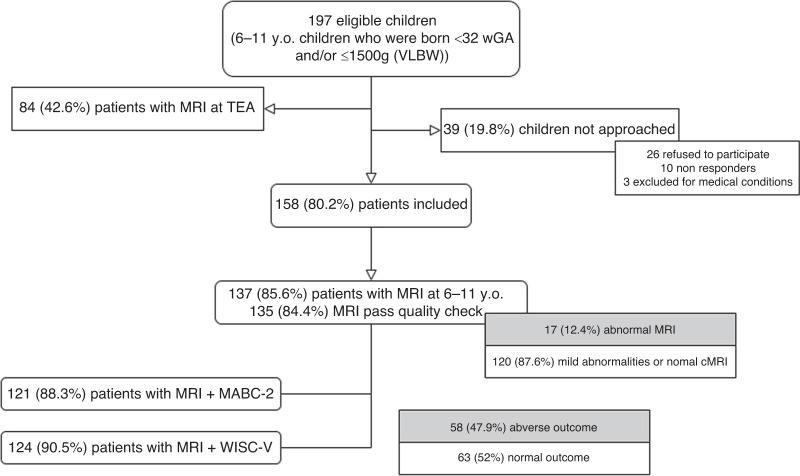
Flowchart of prospective inclusion and final sample size of the study. IQ intelligence quotient, MABC-2 Movement Assessment Battery for Children-2, MRI cerebral magnetic resonance imaging, VLBWI very low birth weight infant, GA weeks of gestational age, WISC-V Wechsler Intelligence Scale for Children-V.

During the study period we contacted 197 eligible subjects. We excluded thirty-nine children (19.8%): 26 declined to participate, 10 were lost to follow-up and 3 were excluded for medical conditions. Our final study population included 158 children between 6 and 11 years of age. The inclusion process and final sample size are summarized in Fig. 1. We performed an MRI on 137 (85.6%) of the included subjects, with 120 (87.6%) of them having mild abnormalities or normal conventional MRI. Moderate/severe abnormal MRI findings and/or cranial ultrasound conditions are described in Table 1s (supplementary material). Of those with an MRI, 121 (88.3%) completed motor evaluation and cognitive outcome was assessed on 124 (90.5%). The median value of total MABC-2 score was 7 [IQR 1–15], corresponding to the 16th percentile [IQR 0.1–95] and the mean FSIQ value was 93.1 (SD 12.7). A detailed description of the scores obtained in all the subtests of MABC-2 and WISC-V is included in Table 2s in the supplementary material.

Those with an adverse outcome (*n* = 58 (47.9%)) compared to those with a normal outcome (*n* = 63 (52.1%)) were born with lower birth weight (1165 grams [IQR 630–2345] vs 1325 grams [IQR 550–2120]; *p* = 0.01). No other clinical or demographic characteristics of the studied participants differed significantly between groups (Table 1).

**Table 1:** Characteristics of the cohort classified by the normal or adverse outcome.

*N* (%) or median [IQR]	Normal outcome *N* = 63	Adverse outcome *N* = 58	Total *N* = 121	*p* value
Female sex	27 (42.9)	33 (56.9)	60 (49.6)	0.94
Birth weight (g)	1325 [550–2120]	1165 [630–2345]	1275 [550–2345]	0.01*
Gestational age (w)	30.3 [24–32.9]	29.3 [25.6–32.7]	30 [24–32.9]	0.13
Age at MRI (years)	8.3 (1.0)	8.4 (1.2)	8.4 (1.1)	0.66
Apgar 1 min	7 [0–9]	6 [3–9]	7 [0–9]	0.01*
Apgar 5 min	8 [4–10]	8 [4–10]	8 [4–10]	0.05*
Bronchopulmonary dysplasia	6 (10)	8 (14.8)	14 (12.2)	0.43
Patent ductus arteriosus	10 (16.7)	12 (22.2)	22 (19.3)	0.45
Sepsis	17 (26.9)	19 (32.8)	36 (29.8)	0.48
Necrotizing enterocolitis	0	2 (3.4)	2 (1.6)	0.27
Retinopathy of prematurity	14 (22.9)	13 (24.1)	27 (23.5)	0.88
Maternal level of education				
Low	23 (33.8)	23 (33.8)	46 (33.8)	
Medium	20 (29.4)	23 (33.8)	43 (31.6)	0.82
High	25 (36.8)	22 (32.3)	47 (34.6)	
CC measurements at school age				
Length (mm)	69.1 (4.8)	67.3 (5.1)	68.2 (5.0)	0.03*
Height at genu (mm)	10 [5.8–13.7]	10 [3.7–14.4]	10 [3.7–14.4]	0.71
Height at body (mm)	5.3 [3.5–7.5]	5.3 [1.8–7.5]	5.5 [1.8–7.5]	0.95
Height at isthmus (mm)	3.6 (0.8)	3.6 (0.9)	3.6 (0.9)	0.49
Height at splenium (mm)	10.2 (2.1)	9.4 (2.3)	9.8 (2.2)	0.02*
Total area (mm^2^)	513.5 (79.0)	481.7 (101.0)	498.3 (91.3)	0.03*
Anterior area (mm^2^)	172.2 (28.2)	165.2 (32.1)	168.9 (30.2)	0.10
Posterior area (mm^2^)	151.7 [60.1–224.1]	143.4 [31.5–202.2]	146.9 [31.5–224.1]	0.11
Central area (mm^2^)	191.7 (34.6)	178.8 (47.4)	185.5 (41.6)	0.04*
DTI measurements				
FA at genu	849 [626–955]	838 [553–951]	843 [553–955]	0.65
FA at splenium	895 [680–1000]	860.5 [342–1000]	881 [342–1000]	0.03*
Neonatal MRI brain injury score^a^ (*n* = 63)	***N*** **=** 30	***N*** = 28	***N*** **=** 58	
Total	2 [0–10]	1 [0–12]	1 [0–12]	0.50
White matter	1 [0–6]	1 [0–6]	1 [0–6]	0.72
Gray matter	0 [0–6]	0 [0–8]	0 [0–8]	0.09

*CC* corpus callosum, *DTI* diffusion tensor imaging, *FA* fractional anisotropy, *IQR* interquartile range. Statistically significant *p*-values are in bold.

*p < 0.05.

^a^Kidokoro et al.^21^

### School-age corpus callosum measurements related to perinatal characteristics

GA was associated with CC length (β = −0.794; *p* = 0.01) and isthmus thickness (β = 0.123; *p* = 0.01) and birth weight was related to total and segmented areas and CC length (Table 2). We found no association of sex and maternal level of education to school age CC measurements.

**Table 2:** CC measurements at school-age that could be predicted by gestational age and birth weigth. adjusted by age at MRI.

Predictor	Gestational age		Birth weight		Total model *p* value	
Variable	β coefficient	*p* value	β coefficient	*p* value	R^2^ adj	*p* value
Length (mm)	−0.794	0.01*	0.005	0.01*	0.08	0.01*
Isthmus (mm)	0.123	0.01*	0.001	0.07	0.18	0.01*
Total area (mm^2^)	−4.551	0.34	0.107	0.01*	0.16	0.01*
Anterior area (mm^2^)	−1.992	0.21	0.029	0.01*	0.06	0.01*
Posterior area (mm^2^)	0.306	0.86	0.028	0.01*	0.08	0.01*
Central area (mm^2^)	−2.865	0.19	0.050	0.01*	0.11	0.01*

Only measurements that were statistically significant are presented.

*P < 0.05.

### Corpus callosum measurements at school age and global outcome

At school age, two linear CC measurements were different between groups of normal and adverse outcomes: height at splenium (10.2 mm (SD 2.1) vs. 9.4 mm (SD 2.3); respectively *p* = 0.02) and total CC length (69.1 mm (SD = 4.8) and 67.3 mm (SD 5.1), *p* = 0.02). Some of the CC areas were smaller among those with adverse outcomes with a total area of 513.5 mm^2^ (SD 79) vs 481.7 mm^2^ (SD 101), *p* = 0.03 and central area of 191.7 mm^2^ (SD 34.6) vs 178.8 mm^2^ (SD 47.4), *p* = 0.04. When excluding those patients with moderate/severe abnormalities in cUS or MRI (those included in Table 1s) we found no major changes to our results except for measurements of total and central areas of the CC, which would not be associated to adverse outcome in the absence of moderate/severe brain injury (Table 3s).

We observed higher FA values for the splenium in those with normal outcomes compared to those with adverse outcomes (895 [IQR 680–1000] vs 860.5 [343–1000]) (Table 1). FA at splenium and height at splenium showed similar results when accounting for GA, birth weight and age at MRI (Table 4s in supplementary material). Age at MRI (mean 8.3 years (SD 1.1)) was not different between outcome groups (*p* = 0.66).

When comparing different subsets obtained, those including measurements of the posterior part of CC (height at splenium: β coefficient = −0.07; *p* = 0.55 and FA at splenium: β = −0.01; *p* = 0.03; model *p* = 0.02) showed higher specificity (83.3%, AUC: 0.65) related to school-age global outcome.

### Corpus callosum subdivision and subscales of motor and cognitive function

Each cognitive and motor subtest of WISC-V and MABC-2 and their relationship with CC measurements were studied (Fig. 3 and Table 3). Accounting for birth weight, GA, sex, and age at MRI we found that CC length and height at splenium were related to manual dexterity (β = 1.04, *p* = 0.01; and β = 2.23, *p* = 0.05, respectively) and with most of the cognitive subscales. Height at genu was also related to some of the cognitive subtests. Length of the CC and height at splenium were the only linear measurements related to the total MABC-2 score (β = 1.10, *p* = 0.05; and β = 2.61, *p* = 0.03, respectively). Both parameters were also related to FSIQ (β = 0.81, *p* = 0.01; and β = 1.94, *p* = 0.01).

**Table 3:** CC measurements related to motor and cognitive subscales.

		Length (mm)	Height at genu (mm)	Height at body (mm)	Height at isthmus (mm)	Height at splenium (mm)	Total area (mm^2^)	Anterior area (mm^2^)	Posterior area (mm^2^)	Central area (mm^2^)	FA at genu	FA at splenium
Motor	Total M-ABC percentile	β = 1.07 *p* = 0.05 Total *p* = 0.05				β = 2.61 *p* = 0.03						
	Manual dexterity percentile	β = 1.04 *p* = 0.01 Total *p* = 0.05				β = 2.18 *p* = 0.05			β = 0.18 *p* = 0.05 Total *p* = 0.05			
	Aiming and catching percentile											
	Balance percentile											
Cognitive	Verbal comprehension score	β = 0.72 *p* = 0.01 Total *p* = 0.01	β = 1.77 *p* = 0.02 Total *p* = 0.01			β = 1.60 *p* = 0.01 Total *p* = 0.01			β = 0.09 *p* = 0.02 Total *p* = 0.01			
	Visuospatial score	β = 1.03 *p* = 0.01 Total *p* = 0.01				β = 1.73 *p* = 0.01 Total *p* = 0.01	β = 0.04 *p* = 0.01 Total *p* = 0.01	β = 0.13 *p* = 0.01 Total *p* = 0.01	β = 0.12 *p* = 0.01 Total *p* = 0.01			
	Fluid reasoning score					β = 1.31 *p* = 0.01 Total *p* = 0.01			β = 0.10 *p* = 0.01 Total *p* = 0.01			
	Working memory score					β = 2.00 *p* = 0.01 Total *p* = 0.01			β = 0.12 *p* = 0.01 Total *p* = 0.01			
	Processing speed score	β = 0.76 *p* = 0.03 Total *p* = 0.01	β = 2.15 *p* = 0.01 Total *p* = 0.03			β = 2.20 *p* = 0.01 Total *p* = 0.01	β = 0.04 *p* = 0.01 Total *p* = 0.02		β = 0.14 *p* = 0.01 Total *p* = 0.01			
	Full-Scale Intelligence Quotient	β = 0.81 *p* = 0.01 Total *p* = 0.01	β = 1.82 *p* = 0.01 Total *p* = 0.01			β = 1.94 *p* = 0.01 Total *p* = 0.01	β = 0.03 *p* = 0.01 Total *p* = 0.01	β = 0.08 *p* = 0.03 Total *p* = 0.01	β = 0.13 *p* = 0.01 Total *p* = 0.01			

All models were adjusted by GA at birth, birth weigth, sex and age at MRI. Only statistically significant results were expressed in the tables.

Total p: p of the full model, including adjusting variables,

*CC* corpus callosum, *GA* gestational age, *MRI* magnetic resonance imaging.

CC posterior area showed a relationship with all dimensions of cognitive function in WISC-V (Fig. 4 and Table 3).

We found no relationship between FA in the genu or in the splenium with any of the motor or cognitive subscales.

### Corpus callosum at term equivalent age and long-term outcome

We retrospectively reviewed the available clinical MRI at TEA of 84 patients from this cohort. The clinical, neuroimaging, demographic and long-term outcome data of this subgroup are detailed in Table 5s. Accounting for GA and birth weight, only length of CC was related with all the long term motor outcomes (Fig. 1s in supplementary material). Height at genu showed relation with manual dexterity and with FSIQ. Total area and posterior area of the CC at TEA were also related with manual dexterity at school age (Fig. 1s).

## Discussion

In this study of children born very preterm and assessed at elementary school age, we found smaller CC measurements related to adverse outcomes with total length of CC and splenium region size related to both cognitive and motor function. Our study showed a significant relationship of the posterior region of the CC with manual dexterity and all cognitive domains in school-age VPT. This finding could be related with impaired white matter development after preterm birth affecting tracts crossing this region of the CC that links extensive areas of sensory and motor integration, being fundamental in tasks such as bimanual function and multiple aspects of cognition.

**Fig. 2:**
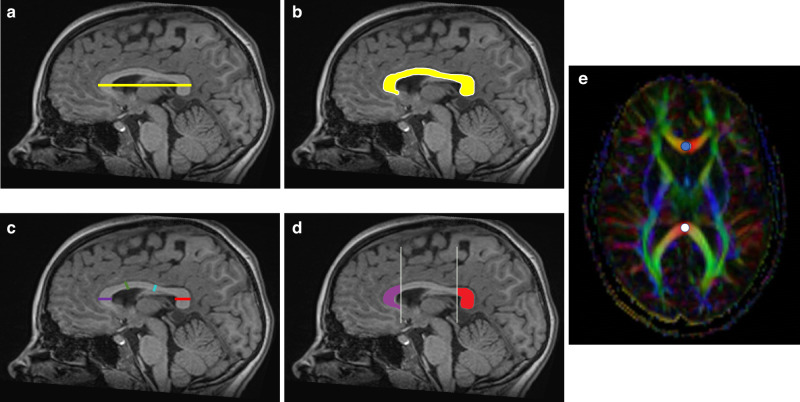
Linear measurements, areas and fractional anisotropy (FA) of corpus callosum. **a** length; **b** total area; **c** linear measurements of genu (purple), body (green), isthmus (light blue) and splenium (red) (performed according to ref. ^33^. **d** anterior and posterior subdivision areas. **e** Region of interest FA measurement at the genu (blue dot) and splenium (white dot).

The CC is an easily measurable structure reflecting brain connections, brain volume and white matter volume.^4–6,23,45^ In VPT, CC altered biometry should not be considered as an isolated disruption in its development as a consequence of preterm birth. However, this anatomical and microstructural alteration of the CC could be part of the spectrum of encephalopathy of prematurity, which includes global WMI. While easy to visualize on the midsagittal plane and easy to measure, no discernable anatomical boundaries divide CC segments. For this reason, there are many proposed subdivision schemes.^27^ Based on models which used DTI and connectivity maps,^27,38^ and considering the well-known relative importance of anterior and posterior regions, we performed a five-subdivisions model (Fig. 2) similar to Delacoste,^39^ Shin^40^ and according to Westerhausen.^41^ This approach is intended to be easily reproducible in clinical practice and to show results congruent with CC connectivity. In this age range, CC biometry remains relatively stable,^35,46^ even in VPT children.^26,47^

Altered patterns of total white matter volume and thinning of CC are common findings in preterm infants^48–52^ with a global reduction of the CC size in children who were born preterm, mostly in the splenium.^24,30,53^ Our findings are in line with previous studies, with gestational age and weight at birth being strongly related to CC length and areas at elementary school age (Table 1).^23,24,50^ CC biometry in these subjects is related with adverse late motor,^3,51^ and neuropsychological outcomes,^23,24,50^ and the splenium is the specific region that correlates the most with cognition.^23,54,55^ Our sample size has allowed us to depict different outcomes of VPT infants relating to the size of the CC, with previous studies mostly having focused on the comparison with healthy term-born children. While smaller CC has been previously demonstrated, the impact of preterm birth on different areas and related to the outcome has not been previously addressed. Our results show that the posterior part of the CC, measured as height at splenium, posterior area, and FA at splenium, is related to adverse prognosis highlighting the importance of this region in motor and cognitive pathways. The splenium of the CC is involved in transference of axons that are related to language, visuospatial integration, complex cognitive functions, behavior and consciousness.^46^ Accordingly, Luders et al.^56^ related the posterior part of the CC and some regions of the anterior part of the CC with total brain volume and FSIQ.^56^ Regional brain volume studies in children born preterm demonstrated reduced white and gray matter volumes in sensorimotor and parieto-occipital regions in preterm compared with full-term infants.^41^ Our findings may underline the relative importance of the splenium in cognitive function and all its subsets in a high-risk neurological population.

Considering the approach based on measuring areas of CC on the mid-sagittal plane, all areas were related to overall prognosis. However, this relationship did not hold when adjusting for GA, sex, birth weight and age at MRI. Although linear measurements of the CC have customarily been used in WMI classification,^17,21,22^ an area-based approach could provide data with higher predictive value and/or more complex information. Correlation between CC segmental areas and cognitive processes in elementary school-age children have been demonstrated in previous studies.^57^ However, there are not many similar studies in children who were preterm.^23,24,50,58^ more studies are needed to assess the usefulness of measures of CC areas at full-term age as long-term prognostic predictors.

During early life and at school age, children who were born VPT have lower FA values when compared to those born at term.^30,59–61^ Furthermore, children born VPT display a lower FA in CC attributable to white matter development disruption in the context of WMI.^28^ FA is also reduced in non-preterm children with thickened corpus callosum due to other conditions.^31^ Our results are in line with previous studies that found an inverse relation between FA assessed by DTI, and motor and cognitive impairment, with splenium being one of the most important regions.^55,62^

The association between CC abnormalities and fine motor and bimanual coordination is well-known.^27,63,64^ In our study, length of CC and height at splenium in school age were related to manual dexterity. On the same way, in the subgroup of patients who had an MRI at TEA, total and posterior areas and length of CC were consistently related with manual dexterity. Similar results, highlighting the importance of the CC in general, and splenium in particular, were obtained both in healthy adolescents^65^ and children with cerebral palsy.^66^ Further research, is warranted to unravel what measurements of the corpus callosum in VPT in the neonatal period may serve as long term predictor of fine motor and bimanual coordination.

**Fig. 3:**
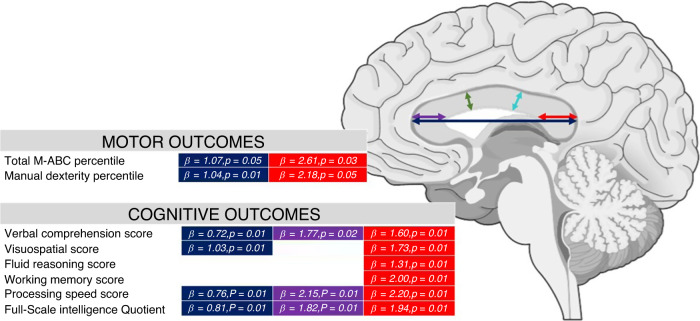
Linear CC measurements related with motor and cognitive subscales. All models were adjusted by GA at birth, sex and age at MRI. Only statistically significant results were expressed in the tables. CC corpus callosum, GA gestational age, MRI magnetic resonance imaging.

**Fig. 4:**
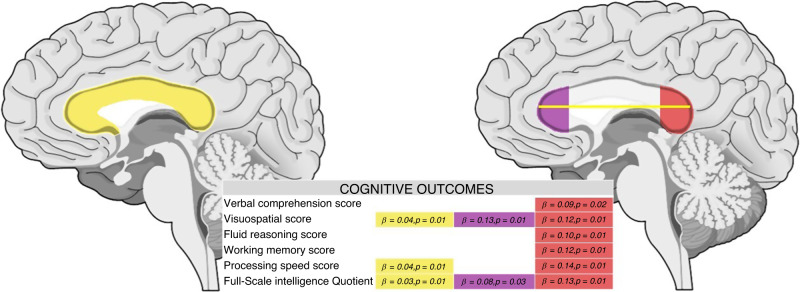
CC areas related with motor and cognitive subscales. All models were adjusted by GA at birth, sex and age at MRI. Only statistically significant results were expressed in the tables. CC corpus callosum, GA gestational age, MRI magnetic resonance imaging.

Many of the CC measurements were related to WISC-V subtests, with total length, height at splenium, total area and posterior area impacting the most on cognitive outcome. However, contradictory findings have been reported relating to regional division and cognitive function. Hutchinson et al.^67^ found smaller posterior areas of the CC in those subjects with higher FSIQ. Nevertheless, this study was based on healthy young adults (mean age 19.2 years old) whereas an accelerated growth pattern of the CC has been described in those who were VPT.^47,68^ Except for this single study, our results are in line with other studies where CC subregions were found to be directly related to FSIQ and WISC-V subscales.^24,47,54,55,69^ It should be noted that parameters measuring the entire CC (length and total area) and posterior region (height at splenium and posterior area) at school age are broadly related to intellectual function. We also found that genu at TEA and at school-age was related to FSIQ, but not with long-term motor performance. Interestingly, the relationship of genu with FSIQ was demonstrated at TEA and at school age (Figs. 3 and 4).

Interestingly, despite being suggested as a valid measure in some neuroimaging studies^17,21,22^ during the neonatal period, we found that the height of the corpus callosum measured at the body and central areas of the CC at elementary school age, were not statistically significant related to any of the cognitive or motor outcomes. We also found no relationship between height at body and an overall adverse prognosis.

While this is one of the studies with a larger sample size of CC biometry in school-age children who were VPT, it has certain limitations that should be addressed. We did not perform a comparison with term-born children as we did not have a control group. However, our aim was to study the differences in CC growth among VPT and our large sample size has allowed us to successfully address this objective. Another limitation of our study is the method of acquiring FA, as other methods, such as track-based spatial statistics have proven to be superior to region of interest (ROI) measurement.^70^ However, while other ROI in different areas are more troublesome, ROI in the CC has a high interrater agreement.^71^

CC biometry in school-age children who were VPT is related with both cognitive and motor outcomes. Length and splenium measurements appear to be good biomarkers of neurological outcome in this population. Future studies are needed to demonstrate the usefulness of these measures in the neonatal period as the most reliable, within the study of the CC as a white matter biomarker.

## Supplementary information

Supplementary Information

## Data Availability

The datasets generated during and/or analysed during the current study are available from the corresponding author on reasonable request.
