# Supplementary material for: Corpus callosum long-term biometry in very preterm children related to cognitive and motor outcomes
Source: Pediatr Res. 2024 Jan 15;96(2):409–17. doi: 10.1038/s41390-023-02994-4 (PMC11343715; doi:10.1038/s41390-023-02994-4)
Supplement: Supplementary file 1 — Supplementary Information [file 41390_2023_2994_MOESM1_ESM.pdf]

Table 1s

**Abnormal MRI finding**	**Participants (n)**
Periventricular cystic leukomalacia	8
Post-hemorrhagic ventricular dilatation (ventriculoperitoneal shunt)	3
Chronic ischaemic/haemorrhagic lesions (porencephaly)	4

Table 1s. Supplementary material. Characteristics of the MRI classified as abnormal.

MRI: magnetic resonance imaging.

Table 2s

MABC-2	Total score	Manual dexterity	Aiming and catching	Balance
**Normal outcome (N= 64)**	10 [7-15]	9.9 (2.5)	10 [3-15]	11 [2-15]
**Abnormal outcome (N=57)**	5 [1-9]	5.2 (2.6)	7 [1-12]	6 [1-12]
**Total (N= 121)**	7 [1-15]	7.7 (3.5)	8 [1-15]	9 [1-15]

WISC-V	VC	VE	FR	WM	PS	FSIQ
**Normal outcome (N= 66)**	101.2 (12.1)	101.3 (11.8)	98.7 (12.0)	100.2 (13.6)	98.7 (11.3)	100.0 (10.0)
**Abnormal outcome (N=58)**	89.5 (12.9)	88.7 (1.3)	87.2 (1.7)	87.1 (11.7)	89.3 (13.7)	85.2 (10.7)
**Total (N= 124)**	95.8 (13.7)	95.4 (14.0)	93.32 (12.7)	94.0 (14.3)	94.3 (13.3)	93.1 (12.7)

Table 2s. Supplementary material. Scoring of MABC-2 and WISC-V. MABC-2 expressed in scalar punctuation. FR: fluid reasoning; FSIQ: full scale intelligence quotient; MABC-2: Movement Assessment Battery for Children Second Edition; PS: processing speed; VC: verbal comprehension; VE: visual spatial; WISC-V: Weschsler Intelligence Scale for Children Fifth Edition; WM: working memory.

Table 3s

N (%) or median [IQR]	Normal outcome N= 57	Adverse outcome N= 49	Total N=106	p value
CC measurements at school age				
Length (mm)	69.0 (4.8)	67.2 (5.2)	68.2 (5.1)	**0.03***
Height at genu (mm)	10.1 [7.1-13.7]	10.1 [4.8-14.4]	10.1 [4.8-14.4]	0.80
Height at body (mm)	5.4 [3.8-7.5]	5.5 [1.8-7.5]	5.5 [1.8-7.5]	0.66
Height at isthmus (mm)	3.6 (0.8)	3.7 (0.8)	3.7 (0.8)	0.75
Height at splenium (mm)	10.5 (1.6)	9.9 (1.6)	10.2 (1.6)	**0.03***
Total area (mm^2)	517.2 (75.6)	499.1 (86.5)	508.9 (80.9)	0.12
Anterior area (mm^2)	173.0 (27.1)	167.3 (30.2)	170.3 (28.6)	0.15
Posterior area (mm^2)	151.7 [94.1-211.4]	146.3 [47-202.2]	147.8 [47-211.4]	0.47
Central area (mm^2)	194.3 (34.6)	185.6 (44.8)	190.3 (39.7)	0.13
DTI measurements				
FA at genu	854 [669-955]	838.5 [713-951]	846 [669-955]	0.94
FA at splenium	899 [750-1000]	862.5 [342-995]	890 [342-1000]	**0.05***

Table 3s. Supplementary material. Relation between CC measurements and normal or adverse outcome. **excluding** patients with moderate/severe abnormalities in cUS or MRI in neonatal period. CC: corpus callosum; DTI: diffusion tensor imaging; FA: fractional anisotropy; IQR: interquartile range. a Kidokoro et al.(21) *p < 0.05

Table 4s

CC measurement	Beta coefficient (β) *P* value	GA β *P* value	Age at MRI β *P* value	Birth weight β *P* value	Total *P* value
Length (mm)	-0.06<br>0.14	-0.03<br>0.13	0.07<br>0.68	-0.01<br>0.18	0.08
Height at genu (mm)	-0.03<br>0.80	0.02<br>0.85	0.05<br>0.75	-0.01<br>0.06	0.19
Height at body (mm)	-0.02<br>0.89	0.02<br>0.87	0.09<br>0.73	-0.01<br>0.06	0.19
Height at isthmus (mm)	0.23<br>0.32	-0.01<br>0.99	0.09<br>0.63	-0.01<br>0.04	0.13
Height at splenium (mm)	-0.16<br>**0.07***	0.04<br>0.76	0.03<br>0.85	-0.01<br>0.06	**0.05***
Total area (mm2)	-0.01<br>0.27	0.01<br>0.96	0.07<br>0.68	-0.01<br>0.16	0.12
Anterior area (mm2)	-0.01<br>0.54	0.01<br>0.91	0.05<br>0.75	-0.01<br>0.09	0.17
Posterior area (mm2)	-0.01<br>0.23	0.02<br>0.87	0.06<br>0.71	-0.01<br>0.12	0.11
Central area (mm2)	-0.01<br>0.32	0.01<br>0.99	0.08<br>0.62	-0.01<br>0.16	0.13
FA at genu	-0.01<br>0.81	-0.01<br>0.99	0.07<br>0.69	-0.01<br>0.11	0.30
FA at splenium	-0.01<br>**0.03***	0.07<br>0.57	0.09<br>0.51	-0.01<br>0.07	**0.02***

Table 4s. Supplementary material. Association of CC measurements and outcome, accounting for GA, age at MRI, birth weight, and sex. FA: fractional anisotropy; GA: gestational age; MRI: magnetic resonance imaging.

Table 5s

N= 84	N (%) or median [IQR]
Female sex	43 (51.2)
Birth weight (g)	1155 [500-1910]
Gestational age (w)	29.0 [24-32.5]
Neonatal MRI brain injury scorea	2 [0-23]
Age at motor and cognitive assessment (y)	8.2 (1.2)
CC measurements at term equivalent age	
Length (mm)	45.4 (3.8)
Height at genu (mm)	2.4 [1.6-6.1]
Height at body (mm)	1.6 [1-3.3]
Height at isthmus (mm)	1.7 (0.4)
Height at splenium (mm)	3.4 [1.2-5.9]
Total area (mm^2)	118.3 (28.0)
Anterior area (mm^2)	33.6 (8.6)
Posterior area (mm^2)	33.9 [15.3-87.5]
Central area (mm^2)	49.9 (13.0)
Motor outcomes	
MABC-2 total score	16 [0.1-91]
Manual dexterity	16 [0.1-91]
Aiming and catching	25 [0.1-95]
Balance	37 [0.1-95]
Cognitive outcomes	
Full scale intelligence	91.9 (13.6)
Verbal comprehension	93.5 (13.9)
Visual spatial	96.1 (13.5)
Fluid reasoning	94.6 (13.3)
Working memory	93.9 (14.7)
Processing speed	92.0 (14.5)

Table 5s. Supplementary material. Characteristics of subgroup of patients with MRI at TEA. CC: corpus callosum; IQR: interquartile range. a Kidokoro et al.(21) *$p < 0.05$
